# Amphibian-Derived Natural Anticancer Peptides and Proteins: Mechanism of Action, Application Strategies, and Prospects

**DOI:** 10.3390/ijms241813985

**Published:** 2023-09-12

**Authors:** Qian Chen, Jing Wu, Xiang Li, Ziyi Ye, Hailong Yang, Lixian Mu

**Affiliations:** Faculty of Basic Medical Sciences, Kunming Medical University, Kunming 650500, China

**Keywords:** amphibians, natural, peptides, proteins, anticancer, mechanism, application

## Abstract

Cancer is one of the major diseases that seriously threaten human life. Traditional anticancer therapies have achieved remarkable efficacy but have also some unavoidable side effects. Therefore, more and more research focuses on highly effective and less-toxic anticancer substances of natural origin. Amphibian skin is rich in active substances such as biogenic amines, alkaloids, alcohols, esters, peptides, and proteins, which play a role in various aspects such as anti-inflammatory, immunomodulatory, and anticancer functions, and are one of the critical sources of anticancer substances. Currently, a range of natural anticancer substances are known from various amphibians. This paper aims to review the physicochemical properties, anticancer mechanisms, and potential applications of these peptides and proteins to advance the identification and therapeutic use of natural anticancer agents.

## 1. Introduction

Cancer has become one of the leading cause of premature death in most countries worldwide. If current cancer incidence trends persist, we predict that by 2070, cancer rates will double from 2020 levels [1], which will create a significant physical and economic burden on people globally. Cancer treatment relies on surgery, radiotherapy, and chemotherapy [2], which have achieved remarkable therapeutic efficacy. However, conventional methods sometimes have some deficiencies, such as lacking a specific drug delivery system and the inability to differentiate between cancerous and normal cells, which may lead to systemic toxicities [3]. Therefore, there is a need for more targeted and effective new drugs to address this issue. In this regard, bioactive substances are increasingly being considered as promising candidates for cancer therapy and applications.

Anticancer substances are abundant in nature, with plants, animals, and microorganisms being major sources [4]. Compared to traditional synthetic small molecule drugs, natural anticancer substances typically have larger molecular weight, more hydrogen bond acceptors and donors, a lower lipid–water partition coefficient, and higher molecular rigidity [5]. Moreover, they can act through multiple pathways and target coordination, rather than a single mode of action. These characteristics significantly expand the range of bioactive compounds and offer promising opportunities for the discovery of active drugs [4]. Recently, amphibians have garnered more attention in the search for biologically active substances due to their ability to survive in diverse habitats, which are attributed to their adaptations in morphology, physiology, biochemistry, and behavior [6]. Amphibians also produce a rich array of chemicals that serve as their self-defense system. In fact, as of June 2023, 108 out of 276 natural anticancer peptides are derived from amphibians (https://aps.unmc.edu/database/anti, accessed on 20 June 2023). Thus, amphibians are a valuable source of bioactive anticancer substances. This paper aims to review the physicochemical properties of natural gene-encoded anticancer substances found in amphibians and to discuss their unique sources, potential mechanisms of action, and future development prospects.

## 2. Physicochemical Properties of Natural Anticancer Peptides and Proteins of Amphibian Origin

The skin of amphibians serves a vital purpose in physiological processes and also acts as a defense against invading pathogens [7]. Additionally, the skin secretions of amphibians contain a diverse range of compounds that possess biologically active functions, such as peptides and esters. In one study, caerin 1.1, derived from Australian tree frogs of the genus Litoria, demonstrated a remarkable ability to suppress the growth of cervical cancer Hela cells. Even at a concentration as low as 1.9 nm, it exhibited significant inhibitory effects [8]. Similarly, BMP1 extracted from Indian toads (Bufo melanostictus Schneider) showed promising results in reducing the MTT value of EAC cells. At a dosage of 1 mg/kg, it achieved a 74.1% reduction, surpassing the effectiveness of 5-FU at 10 mg/kg, which only managed to reduce the MTT value by approximately 64.6% [9]. In the non-small cell lung cancer H157 mouse xenograft tumor model, the tumor growth inhibition rate of 8 mM Dermaseptin-PP (from *Phyllomedusa palliata*) was significantly higher than that of the 0.7 mM cisplatin positive control group. Moreover, there were no observable side effects on mice [10]. However, it is important to consider the differences in drug concentrations when comparing the effects of different drugs. In brief, these compounds have the potential to kill tumors at lower concentrations and with fewer toxic and side effects, which has generated a significant amount of interest.

**Figure 1:**
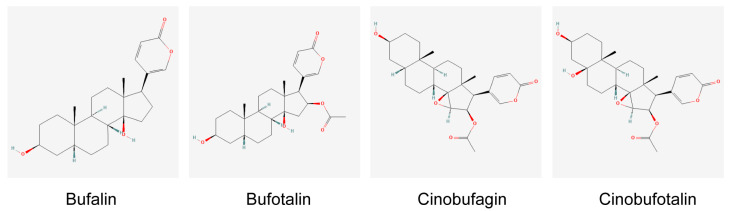
The structure of the main active ingredient in HuaChansu. (Structural formulae from the website: https://pubchem.ncbi.nlm.nih.gov/, accessed on 16 July 2023).

Currently, most peptides with potential anticancer properties have been isolated from the skin of amphibians, although some protease inhibitors obtained from amphibian oocytes have also shown anticancer activity [11]. These peptides, as listed in Table 1, typically consist of 8 to 48 amino acid residues, with a majority bearing a positive charge ranging from +1 to +4, while they are composed of 40% to 70% hydrophobic amino acids [12]. There are also a few examples of anticancer peptides that exhibit negative charge properties. Nuclear magnetic resonance (NMR) studies have shown that these peptides tend to form amphiphilic α-helices in membrane-mimetic solvents, a structure that lays the foundation for their anticancer activity [13]. Another extensively researched compound is HuaChansu, a traditional Chinese medicine isolated from *Bufo gargarizans*, which contains active ingredients such as Bufalin, Bufotalin, Cinobufagin, and Cinobufotalin (Figure 1). Studies have demonstrated its efficacy in treating various cancers, including leukemia, breast cancer, liver cancer, gastric cancer, and colorectal cancer, and it has been shown to possess potent anticancer properties [14].

## 3. Action Mode and Mechanism of Anticancer Peptides and Proteins

Amphibians seem to be a unique source of anticancer substances. From the current literature, it appears that these substances are not only selectively toxic to cancer cells, but also exhibit low toxicity to normal cells [43]. A multitude of anticancer substances derived from amphibian skin have shown an ability to induce cell cycle arrest and apoptosis, and have even demonstrated efficacy in animal models [44].

### 3.1. Interaction Directly through the Cell Membrane

**Figure 2:**
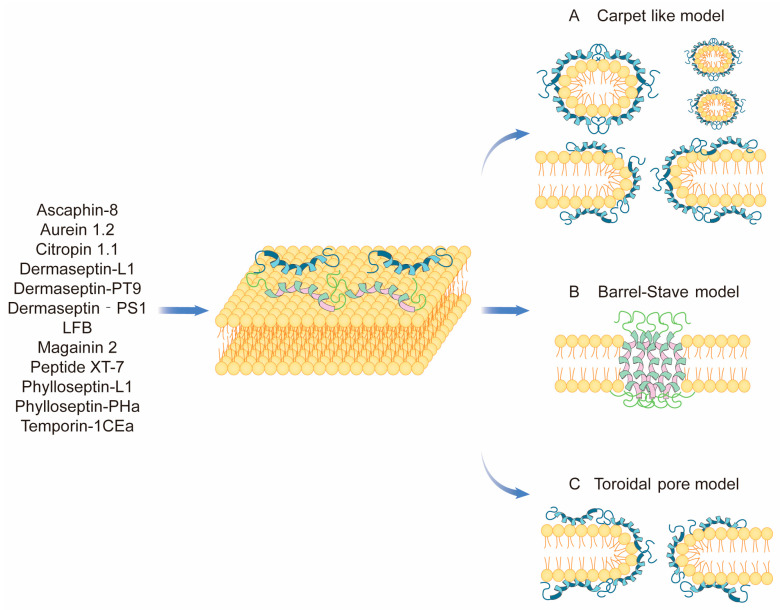
Membrane cleavage of natural anticancer peptides of amphibian origin.

Cancer cells are subject to oxidative stress, inflammation, and other stimuli that cause an increase in glycosylated mucin sialic acid and heparan sulfate on the cell membrane surface [45,46]. This leads to a loss of membrane symmetry and the manifestation of factors such as phosphatidylserine eversion and an increased negative charge on the membrane surface. This property allows positively charged amphiphilic anticancer peptides to selectively interact with cancer cells, leading to the depolarization of their membranes and eventual dissolution. This mechanism also partially explains how anticancer peptides can selectively bind to normal and tumor cells [46]. This direct interaction through the cell membrane is summarized in the following three main models: Carpet-like model, Barrel–Stave model, and Toroidal pore model (Figure 2).

Temporin1CEa is a peptide used by the host to defend against pathogens. It is derived from the skin secretion of the Chinese brown frog (*Rana chensinensis*). When incubated with breast cancer cells at a specific concentration, it causes a loss of membrane symmetry and integrity, leading to a decrease in cell vitality [47]. A similar effect has been observed in melanoma. In fact, studies have shown that human A375 melanoma cells express 50 times more PS than non-cancerous HaCaT cells. As a result, Temporin1CE can bind to PS with high affinity, causing cytotoxicity [48]. Anionic anticancer peptides have also been found to have anticancer effects through direct membrane lysis. Maximin H5 is the first anionic amphibian-derived anticancer peptide demonstrating anticancer activity. After deamination of its C-terminus, Maximin H5 exhibits a lower level of an α-helical structure while penetrating glioma cell membranes, which suggests that the terminal amide group of Maximin H5 is necessary for its membrane-lytic and anticancer activities [49]. Other anticancer peptides with similar functions include citropin 1.1 from the tree frog *Litoria citropa* [50], aurein 1.2 from *Litoria raniformis* [15], and Magainins from *Xenopus laevis* [51] (Table 2).

### 3.2. Acting on Tumor Growth

**Figure 3:**
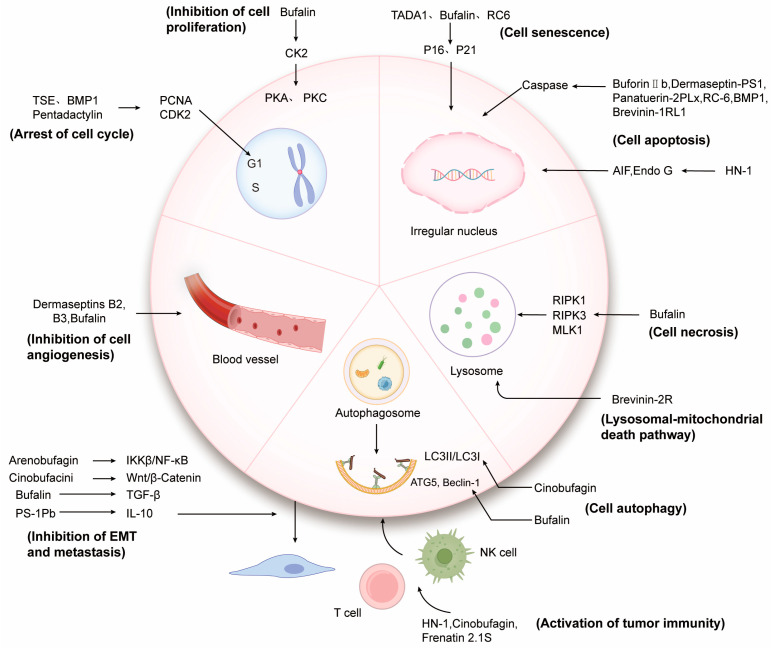
Mechanism of action of natural anticancer peptides and proteins of amphibian origin.

Anticancer substances not only interact directly with cancer cell membranes to alter their integrity and permeability, but they can also target the endogenous pathways of cancer cells through different channels. This can lead to the blockage of the cancer cell cycle, inhibition of cell proliferation, and the induction of cell senescence or death (Figure 3).

#### 3.2.1. Regulation of Tumor Cell Cycle and Proliferation

The cell cycle is a fundamental process in the life of a cell, and its proper functioning relies on the precise regulation of regulatory factors at all levels. When this control is lost, it can lead to cancer. However, the anticancer peptide TSE, derived from the Indian toad *Bufo melanostictus*, has been found to inhibit the expression of PCNA in U937 and K562 cells. PCNA primarily blocks the progression of the G1 phase of the cell cycle and regulates and coordinates DNA replication. As a result, TSE may have therapeutic potential in treating human myeloid leukemia [57]. Pentadactylin is a peptide found in the South American bullfrog *Leptodactylus pentadactylus* that has been shown to be effective in fighting cancer. It works by arresting the cell cycle of B16F10 cells in the S phase and reducing the number of cells in the G1 phase, which significantly inhibits the proliferation of melanoma [58]. In a similar study by Antony Gomes et al., the protein toxin BMP1 was found to arrest the cell cycle of U937 and K562 cells in the sub-G1 and G1 phases. Additionally, it was shown to inhibit the expression of PCNA and CDK2 in HepG2 cells and promote the protein levels of CDKIs such as p21 and p27. These findings suggest that BMP1 can effectively inhibit the proliferation of malignant tumors by regulating the expression of these cell cycle-related genes [9,59]. In leukemia, a similar effect has been noted with Bufalin, which causes an increase in the activity of casein kinase 2 (CK2) by translocating to the nucleus. Additionally, it inhibits the expression of PKA and PKC or binds to β-tubulin, which ultimately leads to the inhibition of proliferation [43].

#### 3.2.2. Inducing Tumor Cells Senescence

Cellular senescence serves as a natural barrier to tumor formation by inhibiting cell proliferation [60]. Therefore, inducing tumor cell senescence may prove to be an effective anticancer strategy. Interestingly, amphibians possess regenerative abilities that are closely linked to aging and cancer. A recent study discovered that the tadpole tail bud base extracts TAD A1 from the stream frog, Strongylopus grayii, which has anticancer potential. Treatment of human malignant embryonic rhabdomyoma cells with TAD A1 resulted in significant morphological changes such as cellular swelling, irregularly enlarged nuclei, and hallmarks of senescent cells. Western blotting results also indicated an increase in the expression of the senescence-related protein p16INK4a [61]. Bufalin is involved in prostate cancer by promoting the expression of senescence-related genes, including CYR6/CCNI, CTGF/CCN2, and the p53 target gene CDKN1A (p21). This leads to an increase in the number of cells undergoing positive senescence, as indicated by β-galactosidase activity [62]. Additionally, certain ribonucleases derived from amphibians [63], such as RC-6 from bullfrogs, have demonstrated anticancer properties. For example, RC-6 has been shown to elevate the levels of the p16 protein in human embryonic carcinoma cells (NT2) and induce cell senescence [64].

#### 3.2.3. Inducing Apoptosis of Tumor Cells

Numerous studies have established that anticancer compounds can activate apoptosis pathways to perform their functions. Apoptosis is classified into two pathways, intrinsic and extrinsic, with caspase-9 and -8 serving as the intermediate activators of both. Additionally, caspase-3, 6, and 7 act as the common apoptosis executors for both pathways [65].

Intrinsic apoptosis is a pathway that occurs in the mitochondria and activates caspases by releasing caspase-activating factors. Recent studies have shown that Pentadactylin not only blocks the cell cycle but also causes cell volume to decrease, mitochondrial membrane potential to decrease, and DNA fragmentation to increase [58]. In addition, Lee et al. discovered that BuforinIIb, an anticancer peptide from the Bufo bufo gargarizans, has a strong killing effect on 62 different cancer cell lines. This peptide can destroy the mitochondrial membrane, release cytochrome C, activate the caspase cascade reaction, induce a series of hydrolyses, and lead to cell disintegration, ultimately initiating endogenous apoptosis [66]. After treatment of U-251 MG cells with Dermaseptin-PS1, there was a significant increase in the protein expression levels of cleaved caspase 3, cleaved caspase 9, apoptosis protease activating factor 1 (Apaf-1), Bcl2-related X protein (Bax), and phosphate p53. Additionally, there was a notable increase in cytochrome c release. Z-VAD-FMK pretreatment was able to abolish the increase in cleaved caspase 3 of U-251 MG induced by Dermaseptin-PS1. These findings suggest that Dermaseptin-PS1 can induce endogenous apoptotic pathways, exhibiting anti-glucose Glioma activity [54]. Several other proteins, including Ranatuerin-2PLx from the pickerel frog [39], ribonuclease RC-6 from *Rana catesbeiana* [64], and protein toxin BMP1 from the Indian toad (*Bufo melanostictus*, *Schneider*) [9,59], are also capable of inducing endogenous apoptosis.

Extrinsic apoptosis, also known as the death receptor pathway, is activated by extracellular signals that trigger intracellular caspase to perform its function. Brevinin-1RL1, an anticancer peptide derived from the *Rana limnocharis*, has been shown to induce significant increases in PI single staining and cleavage of caspase-8, caspase-9, caspase-3, and PARP in lung cancer cells. Additionally, the potential of the mitochondrial membrane decreases, indicating an involvement of both exogenous and endogenous apoptotic pathways [67].

Additionally, specific anticancer peptides may not trigger apoptosis through the caspase-dependent pathway. Instead, they facilitate the transfer of apoptosis-inducing factor (AIF) and endonuclease G (EndoG) from the inner and outer membrane space of mitochondria to the nucleus. This process ultimately leads to DNA cleavage, chromatin condensation, and other effects [68]. One example is the anticancer peptide HN-1, which is derived from the Hainan cascade frog, *Amolops hainanensis*. HN-1 has been shown to activate the MAPK/NF-κB pathway, which in turn boosts the expression of the tumor suppressor gene p53 in cells. This activation also increases the expression of pro-apoptotic proteins from the Bcl-2 family, such as BAX, Bak, and Puma. These proteins can then alter mitochondrial functions, ultimately releasing AIF and EndoG into the nucleus to trigger apoptosis [69].

#### 3.2.4. Inducing Tumor Cells Autophagy

Autophagy is the process by which lysosomes break down intracellular components. It plays a crucial role in maintaining cellular homeostasis by degrading intracellular components and providing cell degradation products [70]. As research on autophagy deepens, its role in tumors has become a topic of widespread interest [71].

After treating U2OS human osteosarcoma cells with Cinobufagin, it was observed that a significant number of autophagosomes were formed, and the conversion of LC3-I to LC3-II was increased [72]. Autophagy is triggered by stresses such as ROS and endoplasmic reticulum stress, which activates pathways such as JNK/P38 [73]. These pathways have been found to be activated in both Cinobufagin-induced osteosarcoma [72] and gastric cancer [74]. Bufalin has been found to promote autophagy in liver cancer, colorectal cancer, and glioma by increasing the ratio of LC3-II/LC3-I [75,76,77]. In liver cancer, the combined use of Bufalin and the autophagy inhibitor 3-MA resulted in a significant reduction in autophagosomes in HCC-LM3 cells and inhibited the expression of LC3-II and Beclin-1 [75]. Conversely, upregulation of LC3-II and p62 inhibited autophagy [75]. Bufalin activated the JNK pathway in colorectal cancer by inducing ROS production in HT-29 cells, leading to increased expression of autophagy-related proteins such as ATG5 and Beclin-1 [76]. Bufalin-induced autophagy is linked to ATP depletion and endoplasmic reticulum stress in glioma. Specifically, Bufalin activates the AMPK signaling pathway by using up ATP, which in turn inactivates mTOR via AMPK-mediated phosphorylation of TSC2 and Raptor. When combined with the endoplasmic reticulum stress inhibitor TUDC, Bufalin transplants GRP78 and LC3-II protein. Knockdown of the eIF2α or CHOP gene or expression of the AMPK/mTOR and PERK/eIF2α/CHOP pathways partially inhibits Bufalin-induced LC3-II transformation, indicating their crucial role in the link between Bufalin-induced autophagy and ER stress [77].

In addition, there are some anticancer substances that can cause both autophagy and apoptosis, such as Bufalin [77], RC-RNase [78], Cinobufagin [72], and so on.

#### 3.2.5. Inducing Tumor Cells Necrosis

In addition to apoptosis and autophagy, cell necrosis is another form of programmed cell death closely linked to tumor patients’ progression and prognosis [79]. Research has demonstrated that targeting tumor cell necrosis can be an effective strategy for avoiding drug resistance in apoptosis and promoting anticancer immunity [80].

Brevinin-1RL1 has been found to not only cause apoptosis in lung cancer cells but also trigger necrosis. Necrosis is characterized by massive vacuolation of cells, increased fragmentation, rupture of cell membranes, and increased release of lactate dehydrogenase (LDH) from intracellular contents [69]. Conversely, Bufalin has been found to induce RIP1-dependent and ROS-dependent programmed necrosis in MDA-MB-468 and T47D cells in breast cancer by targeting the RIP1/RIP3/PGAM5 pathway [81]. The same phenomenon was also observed in drug-resistant triple-negative breast cancer cells, MDA-MB-231 [82]. Research has demonstrated that inhibiting the activity of Caspase-8 can cause cells to transition from apoptosis to necrosis [83]. In the case of glioma, knocking down Caspase-8 resulted in weakened Bufalin-induced apoptosis and a significant increase in cell necrosis. This finding effectively addresses the issue of apoptosis escape without requiring additional treatment [84]. The necrosome, which consists of RIPK1, RIPK3, and MLKL, is the primary executor and defining characteristic of necrosis [85]. Research indicates that partially attenuated necrosis induced by Bufalin can be achieved through the knockout of TNFR1 and RIPK1 genes. In summary, Bufalin can trigger both apoptosis and necrosis in glioma, and their manifestation is closely linked to the status of TNF-α, Caspase-8, and RIPK1 proteins [84].

#### 3.2.6. Inducing Other Forms of Tumor Cells Death

Brevinin-2R is a defensin that has been isolated from the skin of *Rana ridibunda*. This novel peptide has been found to exhibit preferential cytotoxicity against various types of cancer cells, including lymphoma, colon cancer, fibrosarcoma, breast cancer, and lung cancer, as compared to normal cells [86]. Interestingly, Brevinin-2R-induced cell death was found to be independent of caspases, and was associated with decreased mitochondrial membrane potential, increased ROS production, and decreased ATP production. BNIP3 is a protein that can promote apoptosis by targeting mitochondria without requiring caspases. In fact, MCF-7 cells that were transfected with a BNIP3 mutant lacking the transmembrane domain were able to significantly reverse Brevinin-2R-induced cell death [86]. Furthermore, it is crucial to upregulate lysosomal function in response to the high energy demands of tumor hyperproliferation and metastasis [87]. Lysosomal hydrolases, such as cysteine cathepsins B and L, can displace the function of caspases and trigger necrosis or apoptosis. Treatment with Brevinin-2R resulted in enlarged cell lysosomes, and the inhibition of cathepsin B and L partially prevented brevinin-2R-induced cell death. This unique mechanism semi-selectively kills cancer cells through the lysosomal-mitochondrial death pathway [86].

### 3.3. Inhibiting Tumor Angiogenesis

Angiogenesis is often upregulated in malignant tumors, and this process is closely linked to the tumor’s malignant phenotype [88]. As such, inhibiting tumor angiogenesis or the expression of related factors may be an effective strategy for treating tumors.

Dermaseptins B2 and B3 have been shown to effectively inhibit the formation of PC-3 cell colonies and the proliferation of aortic endothelial ABAE cells. In the presence of the angiogenic factor FGF-2, ABAE cells can form a tubular network. However, when combined with Dermaseptins B2 or B3, the inhibition rate of capillary network formation exceeded 88%. This suggests that Dermaseptins B2 and B3 are capable of inhibiting the proliferation and differentiation of vascular endothelial cells [89]. In the context of liver cancer, the combination of Bufalin and Sorafenib has been found to effectively inhibit angiogenesis by regulating the AKT/VEGF signaling pathway [90]. Additionally, Bufalin has been shown to inhibit tumor microenvironment-mediated angiogenesis by down-regulating the expression of pro-angiogenic genes, including VEGF, PDGFA, e-selectin, and p-selectin in HUVECs through the inhibition of the STAT3 pathway. This suggests that Bufalin may have potential as an anti-angiogenic agent in the treatment of colorectal cancer [91] (Figure 3).

### 3.4. Inhibiting Epithelial Mesenchymal Transition and Metastasis of Tumor Cells

Cells that undergo epithelial–mesenchymal transition (EMT) exhibit significantly enhanced migration ability. In epithelial tumors, EMT plays a crucial role in tumor migration, invasion, and metastasis [92]. Studies have demonstrated that NF-κB, a multifunctional transcription factor, can regulate the expression of various target genes during the migration and invasion of tumor cells [93]. The IκB kinase (IKK) complex, an upstream kinase necessary for activating NFκB activity, has been identified as a critical factor in this process. Treatment with Arenobufagin can reverse the EMT process of lung cancer cells by inhibiting the cascade activation of the IKKβ/NF-κB pathway, thereby effectively inhibiting the metastasis of lung cancer [94]. In colorectal cancer, the Wnt/β-Catenin pathway is crucial in promoting tumor growth and metastasis [95]. However, treatment with Cinobufacini has been found to promote the expression of APC, a protein that facilitates the nuclear transfer of β-Catenin [96]. At the same time, the expression of β-Catenin, wnt3a, c-Myc, cyclin D1, and MMP7 is inhibited, indicating that Cinobufacini can effectively inhibit the junctional role of EMT in rectal cancer and its metastasis, by blocking the Wnt/β-Catenin protein signaling pathway [97]. Matrix metalloproteinases are enzymes that can break down the extracellular matrix and facilitate the movement of cancer cells into the basement membrane [98]. Cinobufacin has been shown to inhibit the invasion of pancreatic cancer cells by suppressing the expression of MMP-2 and MMP-9. In addition, in the liver, cinobufacin has been found to reduce the specific gravity and expression of MMP-2 and MMP-9 in liver metastases. These findings suggest that cinobufacin may possess anti-hepatic metastasis properties for pancreatic cancer [99]. TGF-β is known to promote tumor cell invasion and metastasis through several mechanisms, including angiogenesis, inflammation, immune escape, and epithelial–mesenchymal transition [100]. However, bufalin has been found to inhibit TGF-β-induced EMT. In addition, activation of the transcription factor Twist2 and expression of the TGF-β receptor have been shown to suppress metastasis in liver and lung cancers [101]. It is worth noting that bufalin can also promote the expression of metalloproteinase tissue inhibitor protein [102], regulate the phosphorylation of tight junction and invasion-related protein GSK3-β [103], and induce the overexpression of fibronectin [104], among other ways to inhibit tumor cell migration and metastasis.

Furthermore, patients with various tumors have been found to have elevated circulating concentrations of IL-10, which is known to be associated with tumor progression and metastasis [105]. Host defense peptides Ps-1Pb and Ps-2Pa, derived from *Pseudhymenochirus merlini*, have been shown to inhibit the release of IL-10 and have the potential to improve the efficacy of other chemotherapeutic drugs while also inhibiting metastasis. However, the effect of Ps-1Pb and Ps-2Pa on IL-10 and their mechanism of action on cancer cell metastasis require further exploration [105] (Figure 3).

### 3.5. Activating Tumor Immunity

Tumor cells exist within a complex tumor microenvironment. On one hand, the tumor cells can promote growth and metastasis by secreting transforming growth factor-β, vascular endothelial growth factor, and other factors. However, they also have various mechanisms, such as surveillance functions, that enable them to evade the immune system [106]. Consequently, targeting immune attacks and breaking immune escape has become a major area of research. Active substances secreted by amphibians are a crucial component of the innate immune defense system and can regulate various biological processes, including inflammation, injury, and even tumors.

In addition to inducing tumor cell apoptosis, HN-1 also has the ability to regulate cancer immune-related cytokines/chemokines such as IL-12 and MCP-1 and activate T lymphocytes, B lymphocytes, monocytes, and macrophages. The activation of CD4+ T cells leads to a significant increase in their proliferation as well as the activation of tumor-associated macrophages, resulting in an increased CD4+/CD8+ ratio and enhanced tumor immunity [69]. Similarly, Cinobufagin has been shown to stimulate the proliferation of splenocytes, enhance the phagocytosis of peritoneal macrophages, and promote the production of Th1-related cytokines interferon-γ and tumor necrosis factor-α while inhibiting the levels of Th2 cytokines interleukin-4 and interleukin-10. This leads to an increased Th1/Th2 ratio and has the potential to activate immunity in cancer [107]. Furthermore, Cinobufocini has the ability to inhibit TNF-α-induced inflammatory activation of NF-κB and COX-2 in lung adenocarcinoma cells, thereby exerting anticancer effects [108]. Natural killer cells, a crucial class of innate immune cells, perform tumor recognition and killing functions by regulating the balance of activating and inhibitory receptors without attacking healthy self-tissues [109]. Upon injection of Frenatin 2.1S, there was a significant increase in CD3-CD49b+ NK cells in the peritoneal cavity, leading to the cytotoxicity of NK cells through the mediation of Fas ligand (FasL) expression, resulting in cell lysis and the promotion of immune regulatory factor IL-10 production [110] (Figure 3).

### 3.6. Other Possible Ways to Relate to Oxidative Stress

Imbalance of redox homeostasis is a common characteristic in most cancers, which tend to maintain a high level of oxidation. This oxidative environment promotes both anti-oxidation and pro-oxidation therapeutic strategies [111]. The body’s production of reactive oxygen species (ROS) plays a crucial role in this process [111]. Research has demonstrated that ROS can participate in various stages of tumor development, including proliferation, invasion, metastasis, and angiogenesis. Additionally, ROS is closely associated with chronic inflammation, cell cycle regulation, and tumor suppressor gene expression [112]. Amphibian skin is known to engage in respiratory exercise, which also contributes to the regulation of redox levels in the body to some extent [7]. Further investigation is required to determine its potential role in cancer regulation. However, it has been proven that peptides secreted by the skin possess antioxidant effects, and certain peptides can inhibit cancer development by promoting ROS generation [113]. Yao Li et al. discovered that the level of ROS in U251 cells treated with 100 nM Bufalin increased by 4.93 times. Additionally, the ratio of glutathione (GSH) to oxidized glutathione (GSSG) and the adenosine triphosphate (ATP) content decreased significantly. These findings suggest that Bufalin promotes GSH consumption and affects ATP production in U251 cells. However, it remains unclear whether these changes ultimately result in cell apoptosis [113]. Similar results were also observed in Bufalin, which induces autophagy and cell death in human colorectal cancer cells by promoting the generation of ROS and activating JNK [76]. Bufadienolides were found to increase ROS levels in HeLa cells, cause cell cycle arrest in S and G2/M phases, and exhibit cytotoxic effects [114].

As mentioned earlier, although these anticancer substances caused changes in oxidative stress levels, whether it was because oxidative stress ultimately affects tumor progression has not been fully elucidated by the researchers, which is one of the questions that will need to be addressed in subsequent studies.

## 4. Application Strategies of Natural Anticancer Peptides and Proteins from Amphibians

### 4.1. Structural Modification

For decades, amphibians have been a valuable source of natural anticancer substances. However, the stability and bioavailability of these substances are limited, which reduces their effectiveness. To address this issue, structural modifications can be made to these natural compounds. By doing so, their pharmacological properties can be improved, and their efficacy as anticancer drugs can be enhanced. There are two primary methods for modifying subject molecules: the addition of positively charged residues and the incorporation of d-amino acids [115].

Under the condition of keeping the conservative amino acids unchanged, natural anticancer peptides can be altered through the substitution or modification of certain amino acids. This process can effectively reduce immunogenicity and toxicity while also increasing tolerance to proteases and enhancing anticancer activity. Brevinin-1OS (B1OS) was a wild-type des-Leu2 brevinin peptide from *Odorrana schmackeri*. Two variants, B1-OS-L and B1-OS-DL, were designed by adding L-leucine and d-leucine residues to the second-position leucine residues, respectively. It was discovered that their anticancer activity is approximately 10 times greater than B1OS. Interestingly, human non-small cell lung cancer cells cannot survive after 2 h of treatment with only 10 μM of d-leucine alone, significantly increasing the killing efficiency. Furthermore, this treatment showed low cytotoxicity to normal cells [32].

The cationic-enhancing analog t-DPH1-5K was designed using the peptide t-DPH1 from *Phyllomedusa hypochondrialis*. The study discovered that, by introducing two net positive charges, the MIC value of t-DPH1-5K decreased by 7–20 times on PC-3, H838, H157, and U251MG tumor cells [116]. A similar phenomenon was observed in the peptides found in *Phyllomedusa sauvagii*. By substituting two neutral amino acid residues with lysine and leucine residues at positions 10 and 11 in DPS3, we obtain L^10,11^-DPS3, which exhibits a significantly enhanced anticancer effect [117].

The high hydrophobicity of temporins often results in strong cytotoxicity towards normal cells, which presents a significant limitation in their application as anticancer peptides. This issue must be addressed in order to fully utilize their potential [118]. By substituting leucine 4 on the hydrophilic surface, we obtained a new derivative called temporin-PKE-3K. This derivative showed a significant reduction in its toxicity to HACAT cells [115]. The evaluation of six temporin-1CEa-derived peptides’ anticancer activity suggests that increasing cationicity while maintaining moderate hydrophobicity is an effective strategy to improve anticancer cytotoxicity and reduce hemolytic activity [119].

### 4.2. Combined with Other Drugs

**Figure 4:**
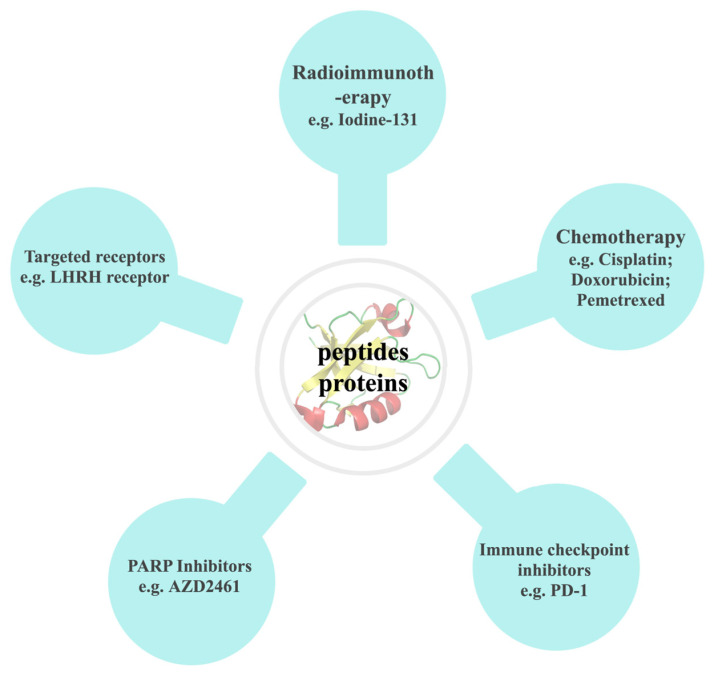
Combination strategies of natural anticancer peptides and proteins.

In recent years, there has been a significant auxiliary effect in the field of cancer treatment through the combination of natural anticancer drugs with advanced technologies and materials (Figure 4). This also has led to the precise control of curative effects and targeting, resulting in improved outcomes in experimental studies and therapeutic effects on patients.

#### 4.2.1. Combined with Radioimmunotherapy

Radioimmunotherapy (RIT) is an effective anticancer strategy established by binding radioisotopes to specific molecules that can recognize and selectively search for cancer cells [120]. In the field of diagnosis and treatment, there are numerous radionuclides utilized. However, iodine-131 stands out due to its longer half-life of 8.1 days and fewer side effects. This makes it a popular choice for clinical radioimmunoconjugates when it comes to radioisotope labeling [121].

In human anaplastic thyroid cancer, Lin Ruoting et al. developed a radioimmunocouple based on caerin 1.1 natural peptide combined with iodine labeling which has been shown to have multiple inhibitory effects on CAL62 cells. It can inhibit the phosphorylation of Akt, arrest cells in the S phase, induce apoptosis, activate immunity, and effectively inhibit the growth and migration of thyroid cancer when used in conjunction with radiotherapy. Further studies are required to determine whether this method is suitable for in situ treatment of anaplastic thyroid cancer [122].

#### 4.2.2. Combined with Chemotherapy

Cisplatin is considered as the pioneer of platinum-based chemotherapeutic drugs, belonging to the first generation. Despite being one of the oldest chemotherapeutic drugs, it still holds a prominent position in clinical practice and is widely used. A meta-analysis showed that, compared with platinum-based chemotherapy (PBC) alone, Huachansu injection (HSC) + PBC significantly improved the 1-year and 2-year survival rates of patients with advanced NSCLC and reduced the adverse reactions of patients. This suggests that HCS may be a valuable adjuvant therapy for PBC in the treatment of advanced NSCLC. However, high-quality and more extensive sample size studies are needed to support this conclusion [123].

In their study, Jufeng Xia et al. discovered that the growth of HCC cells was significantly suppressed when cinobufacini and doxorubicin were used in combination. The combination group induced apoptosis more effectively by influencing the protein and RNA expression of apoptosis-related elements like Bcl-2, Bax, Bid, and cytochrome c. Additionally, as a mixture of components, it exhibited stronger apoptosis-inducing activity than a specific single component or a simple mixture of few components. In conclusion, combining cinobufacini and doxorubicin presents a promising new approach to inhibiting the proliferation of HCC cells [124].

Malignant mesothelioma is cancer that originates from mesothelial cells and is known for its aggressive nature. Unfortunately, there are limited clinical treatment options available for this disease. One promising option is a combination therapy involving pemetrexed and cisplatin, which has been shown to improve response rate, overall survival, and quality of life in mesothelioma patients [125]. However, it is essential to note that many patients treated with this therapy experience tumor progression or recurrence within a year [125]. Recent studies have shown that a sialic acid-binding lectin isolated from Rana catesbeiana oocytes has the potential as an anticancer agent. When tested on H28 cells in combination with pemetrexed, cisplatin, or alone, this lectin was found to decrease cell viability and proliferation levels and arrest the cell cycle. Compared with pemetrexed or cisplatin, cSBL has exhibited potent anticancer effects in various mesothelioma cell lines owing to its high selectivity for cancer cells and cytotoxic activity. The combination of pemetrexed and cSBL has shown a robust synergistic effect, which is similar to or even better than the conventional pemetrexed and cisplatin regimen [126].

#### 4.2.3. Combined with PD-1 Immune Checkpoint Inhibitors

The use of immune checkpoint inhibitors has significantly enhanced the long-term response rates in patients with different types of solid tumors. Nevertheless, when used alone, immune checkpoint inhibitors like PD-12 and CTLA-4 blockade are not as effective in treating advanced cervical cancer [127]. A clinical trial has demonstrated that combining immune checkpoint PD-1 blockade with therapeutic vaccination results in a synergistic effect, leading to effective tumor control. Herein, Guoying Ni et al. demonstrated that administering caerin 1.1/1.9 peptide intratumorally, in combination with E7 antigen-specific immunotherapy and IL-10 and PD-1 blockade, improved the TC-1 tumor microenvironment (TME) and increased the efficacy of anti-PD-1 treatment in TC-1 tumor-bearing mice. The injection of caerin 1.1/1.9 peptides led to an increase in the number of M1 macrophages and NK cells, both of which exhibited elevated levels of immune activation. Additionally, the functions of M2 immunosuppressive macrophages and immunosuppressive B cells were reduced, which prolonged the peptide’s action time [128].

#### 4.2.4. Combined with PARP Inhibitors

The inhibition of Poly (ADP-ribose) polymerase (PARP) is known to cause the accumulation of DNA strand breaks, resulting in cellular damage. As a result, PARP inhibitors (PARPi) have become a promising tool in the fight against various types of cancer [129]. The study found that using onconase with a PARPi, AZD2461, had a low synergistic effect. However, it was discovered that AZD2461 did not induce drug resistance in A375 cells even after being treated with high concentrations of the molecule for 2 months. In contrast, A375 cells that were treated with AZD2461 for a longer period became more susceptible to the pro-apoptotic effect of ONC compared to untreated A375 cells. This study can provide guidance on the clinical use of AZD2461 combination therapy [130].

#### 4.2.5. Binding to Tumor Cell Targeted Receptors

In addition to chemotherapy, androgen deprivation therapy using luteinizing hormone-releasing hormone (LHRH) agonists or antagonists is an alternative treatment option for men with inoperable hormone-sensitive metastatic prostate cancer. However, this approach has not been effective in improving the median survival time of patients [131]. A potential solution to this problem is the use of Dermaseptins B2, a peptide derived from the *Phyllomedusa bicolor*, which is combined with hormonotoxin (H-B2) to target the LHRH receptor on the surface of prostate cancer cells. This optimization of the interaction between the peptide and the cell membrane shows promise in improving treatment outcomes for patients. The study revealed that, while the enrichment of cell surface receptors was not significant, the combination of Dermaseptins B2 with hormonotoxin can effectively reduce the toxic dose of Dermaseptins B2 alone. This finding indicates a promising avenue for targeted therapy through further optimization [132].

## 5. Prospects for the Development of Natural Anticancer Peptides and Proteins from Amphibians

Amphibians, as an ancient group, have the unique ability to adapt to both terrestrial and aquatic life. This evolutionary trait has also led to the development of their distinct adaptive immune and defense systems [133]. Due to their natural resistance to cancer, spontaneous tumors in amphibians are rare, and even in laboratory settings, it is challenging to induce tumors using chemical or biological methods. This natural advantage makes amphibians a promising source for mining anticancer substances [134]. With the advent of new technologies and methods to enhance drug efficacy and promote drug development and clinical transformation, it is expected that more highly effective anticancer substances will be discovered and elucidated. However, continuous efforts are still required to explore these possibilities.

## Figures and Tables

**Table 1:** Information on anti-tumor peptides from amphibians.

Name	Species	Amino Acid Numbers (aa)	Primary Structure	Cancers	References
Aurein 2.5	*Litoria aurea*, *Litoria raniformis*	16	GLFDIVKKVVGAFGSL	Leukaemia, Lung, Colon, CNS, Melanoma, Ovarian, Renal, Prostate, Breast	[15]
Aurein 2.6	*L* *itoria raniformis*	16	GLFDIAKKVIGVIGSL		
Aurein 3.1	*Litoria aurea*, *Litoria raniformis*	17	GLFDIVKKIAGHIAGSI		
Aurein 3.2	*Litoria aurea*, *L itoria raniformis*	17	GLFDIVKKIAGHIASSI		
Aurein 3.3	*L* *itoria raniformis*	17	GLFDIVKKIAGHIVSSI		
Maximin-1	*Bombina maxima*	27	GIGTKILGGVKTALKGALKELASTYAN	Leukaemia, Bladder	[16]
Maximin-3	*B* *ombina maxima*	27	GIGGKILSGLKTALKGAAKELASTYLH	Leukaemia, Bladder	[16]
Maximin-4	*B* *ombina maxima*	27	GIGGVLLSAGKAALKGLAKVLAEKYAN	Leukaemia	[16]
Maximin-5	*B* *ombina maxima*	27	SIGAKILGGVKTFFKGALKELASTYLQ	Leukaemia	[16]
Bombinin H2	*Bombina variegata*	20	IIGPVLGLVGSALGGLLKKI	NSCLC	[17,18]
Temporin A	*Rana temporaria*	13	FLPLIGRVLSGIL	NSCLC	[17,19]
Temporin L	*R* *ana temporaria*	13	FVQWFSKFLGRIL	Leukaemia, Lymphoma	[20]
Temporin-1DRa	*Rana draytonii*	14	HFLGTLVNLAKKIL	Liver	[21]
Temporin-PE	*Pelophylax esculentus*	13	FLPIVAKLLSGLL	Lung, Glioma, Prostate, Breast	[22]
Ps-1Pb	*Pseudhymenochirus merlini*	29	IKIPSFFRNILKKVGKEAVSLIAGALKQS	Lung, Breast, Colon	[23]
Ps-2Pa	*Pseudhymenochirus merlini*	27	GIFPIFAKLLGKVIKVASSLISKGRTE	Lung, Breast, Colon	[23]
Dermaseptin-B2	*Phyllomedusa bicolor*	33	GLWSKIKEVGKEAAKAAAKAAGKAALGAVSEAV	Prostate, Pancreas, Melanoma	[24]
Dermaseptin-B3	*Phyllomedusa bicolor*	29	ALWKNMLKGIGKLAGQAALGAVKTLVGAE	Prostate, Breast	[24]
Dermaseptin-B4	*Phyllomedusa bicolor*	28	ALWKDILKNVGKAAGKAVLNTVTDMVNQ	Breast	[25]
DRS-DU-1	*Phyllomedusa duellmani*	28	ALWKSLLKNVGKAAGKAALNAVTDMVNQ	Lung, Prostate	[26]
Dermaseptin-PD1	*Pachymedusa dacnicolor*	31	GMWSKIKETAMAAAKEAAKAAGKTISDMIKQ	Glioma	[27]
Dermaseptin-PD2	*Pachymedusa dacnicolor*	33	GMWSKIKNAGKAAAKAAAKAAGKAALDAVSEAI	Lung, Prostate, Glioma	[27]
Dermaseptin-PH	*Phyllomedusa hypochondrialis*	23	ALWKEVLKNAGKAALNEINNLVQ	Lung, Glioma, Prostate, Breast	[28]
Dermaseptin-PS4	*Phyllomedusa sauvagii*	28	ALWKTLLKHVGKAAGKAALNAVTDMVNQ	Lung, Glioma, Prostate, Breast	[29]
Brevinin-1GHd	*Hylarana guentheri*	24	FLGALFKVASKLVPAAICSISKKC	Lung, Glioma, Prostate, Breast	[30]
Brevinin-1H	*Amolops hainanensis*	21	FALGAVTKVLPKLFCLITRKC	Lung, Prostate, Melanoma, Colon	[31]
Brevinin-1OS	*Odorrana schmackeri*	23	FPLIASLAGNVVPKIFCKITKRC	Lung, Glioma, Prostate, Breast, Colon	[32]
Bombinin-BO1	*Bombina orientalis*	25	GIGSAILSAGKSIIKGLAKGLAEHF	Liver	[33]
Bombinin H-BO1	*Bombina orientalis*	17	IIGPVLGLVGKALGGLL	Liver	[33]
BLP-7	*Bombina orientalis*	27	GIGGALLSAGKSALKGLAKGLAEHFAN	Liver	[34]
Bombinin H-BO	*Bombina orientalis*	17	IIGPVLGLIGKALGGLL	Liver	[34]
PE-BBI	*Pelophylax esculentus*	16	GALKGCWTKSIPPKPC	Colon	[35]
Phylloseptin-PHa	*Pithecopus hypochondrialis*	19	FLSLIPAAISAVSALANHF	Colon, Breast	[36]
Alyteserin-2	*Alytes obstetricans*	16	ILGKLLSTAAGLLSNL	Lung	[37]
HECI	*Hylarana erythraea*	17	TVLRGCWTFSFPPKPCI	Lung, Prostate, Breast	[38]
Ranatuerin-2PLx	*Rhodopseudomonas palustris*	28	GIMDTVKNAAKNLAGQLLDKLKCKITAC	Lung, Prostate, Breast, Glioma	[39]
Hymenochirin-1B	*Hymenochirus boettgeri*	27	KLSPETKDNLKKVLKGAIKGAIVAKMV	Lung, Breast, Colon, Liver	[40]
Esculentin-2CHa	*Lithobates chiricahuensis*	37	GFSSIFRGVAKFASKGLGKDLAKLGVDLVACKISKQC	Lung	[41]
Figainin 1	*Boana raniceps*	18	FIGTLIPLALGALTKLFK	Melanoma, Breast, Cervical	[42]

**Table 2:** Peptides from amphibians that exert anti-tumor effects by membrane dissolution. (The net charges theoretical values obtained from the prediction of the peptide when it is under neutral conditions (pH = 7), the website is https://www.novopro.cn/tools/calc_peptide_property.html, accessed on 16 July 2023).

Name	Species	Amino Acid Numbers (aa)	Primary Structure	Net Charges	Cancers	References
Ascaphin-8	*Ascaphus truei*	19	GFKDLLKGAAKALVKTVLF	3	Liver	[52]
Aurein 1.2	*Litoria raniformis*	13	GLFDIIKKIAESF	0	Leukaemia, Lung, Colon, CNS, Melanoma, Ovarian, Renal, Prostate, Breast	[15]
Citropin 1.1	*Litoria citropa*	16	GLFDVIKKVASVIGGL	1	Leukaemia, Lung, Colon, CNS, Melanoma, Ovarian, Renal, Prostate, Breast	[50]
Dermaseptin-L1	*Hylomantis lemur*	32	GLWSKIKEAAKAAGKAALNAVTGLVNQGDQPS	2	Liver	[6]
Dermaseptin-PT9	*Phyllomedusa tarsius*	25	GLWSKIKDAAKTAGKAALGFVNEMV	2	Lung, Glioma, Prostate, Breast, Pancreas	[53]
Dermaseptin-PS1	*Paralabidochromis sauvagei*	31	ALWKTMLKKLGTVALHAGKAALGAVADTISQ	3.1	Glioma	[54]
LFB	*Limnonectes fujianensis*	33	GLFSVVKGVLKGVGKNVSGSLLDQLKCKISGGC	3.9	Lung, Breast, Glioma, Colon	[55]
Magainin 2	*Bombina maxima*	23	GIGKFLHSAKKFGKAFVGEIMNS	3.1	Bladder	[51]
Peptide XT-7	*Xenopus tropicalis*	18	GLLGPLLKIAAKVGSNLL	2	Liver	[52]
Phylloseptin-L1	*Hylomantis lemur*	18	LLGMIPLAISAISALSKL	1	Liver	[6]
Phylloseptin-PHa	*Pithecopus hypochondrialis*	19	FLSLIPAAISAVSALANHF	0.1	Lung, Glioma, Prostate, Breast, Colon	[36]
Temporin-1CEa	*Rana chensinensis*	17	FVDLKKIANIINSIFGK	2	Melanoma, Breast, Liver, Lung, Carcinoma, Colon	[47,48,56]

## Data Availability

Not applicable.
